# Supplementary material for: Chromothripsis during telomere crisis is independent of NHEJ, and consistent with a replicative origin
Source: Genome Res. 2019 May;29(5):737–49. doi: 10.1101/gr.240705.118 (PMC6499312; doi:10.1101/gr.240705.118)
Supplement: Supplemental Material [file supp_gr.240705.118_Supplemental_file_1.zip › contigs/annotated_contigs/DB90/contig.2.DB90_length_727_mean_cov_4.1265474553.docx]

**DB90_length_727_mean_cov_4.1265474553**

```
AAGAGTGTCTCTTTACACGATGTACTGCCTGGAATTTTCAAAGGGGTGACTCAGTTGATATAAAACTGCCCGTCCTACTCTCTTTAATG
 >chr9:120780421-120780822 + E=2e-220
CACCTTTTCTGATTTCTGTGCTATGCCCAGGTGCTAAAATCACTCACCTGGTTTCCTTTGCTCTTGTGAAGGTACTTTTGTGTGTTGAT

TGTTGTTCAAATTGATGTTTCTGAAGGGGGAACAGTTGAAAGTCCTATTCCATCATATTGCTCTTCATCAGTATGTTTTATATTTATGA

CCAATGGTTTTAAAATATTGCAGGCATTTTTTTCATGTGTCTTTTGGCTGCATAAATGTCTTCTTTTGAGGAGTGTCTGTTCATATCCT

TTGCCCACGTGTTGATGGGATTGTTTTCTTCTTGTAAATTTG|TTT|TCATCTTAGATAAAATGTTCATTTAGCCACAGAATCTTTGAA
                                           >chr9:120783989-120784318 + E=4e-183
GCTCAGAATTAGAATAAAGTCACTACGACCAATCCCTAGTTCCCTGTGAATTCCACAGTTTCTTGCACAATATTTCAGGCCTGTGGTCA

GCCAGTTAAAGAAACTGTAGTCTCTGGCTTGTAGTTAGGAAATAAGAGAAAAGACCAAAAACCTGACCTATAGAGAACTAAGAGAGCTT

AAAACGCCAGAACCAAATAGACTCCTGATTCTAGGCAGGTATTTGATGGGTCAGAGAGAGAGAAATAAATACTGCAGTAATGATGGCTA

CAATCTCAGAAGTGATT
```
